# Modulating the Spin Seebeck Effect in Co_2_FeAl Heusler Alloy for Sensor Applications

**DOI:** 10.3390/s20051387

**Published:** 2020-03-03

**Authors:** Marcus Vinicius Lopes, Edycleyson Carlos de Souza, João Gustavo Santos, João Medeiros de Araujo, Lessandro Lima, Alexandre Barbosa de Oliveira, Felipe Bohn, Marcio Assolin Correa

**Affiliations:** 1 Departamento de Física, Universidade Federal do Rio Grande do Norte, Natal 59078-900, RN, Brazil; 2 Instituto Federal de Educação Ciência e Tecnologia do Ceará, Quixadá 63902-580, CE, Brazil

**Keywords:** spintronics, CFA, thermoelectric effect, spin seebeck effect

## Abstract

The thermoelectric conversion technique has been explored in a broad range of heat-flow sensors. In this context, the Spin Seebeck Effect emerges as an attractive candidate for biosensor applications, not only for the sensibility improvement but also for the power-saving electronic devices development. Here, we investigate the Longitudinal Spin Seebeck Effect in films with a Co${}_{2}$FeAl/W bilayer structure grown onto GaAs (100) substrate, systems having induced uniaxial magnetic anisotropy combined with cubic magnetic anisotropy. From numerical calculations, we address the magnetic behavior and thermoelectric response of the films. By comparing experiment and theory, we explore the possibility of modulating a thermoelectric effect by magnetic anisotropy. We show that the thermoelectric voltage curves may be modulated by the association of magnetic anisotropy induction and experimental parameters employed in the LSSE experiment.

## 1. Introduction

Presently, the thermoelectric effects, such as the Seebeck effect, are widely explored in the direct conversion of heat-flow in voltage signal [1,2,3]. However, usually, these systems present a high heat resistance, leading to limitations in biosensor applications [4]. On the other hand, thermoelectric effects based on spin dynamics have received increasing attention in recent past, not just to the great technological potential in power-saving electronic devices [5,6,7,8,9], but also for photodetectors [10,11], diode [12], and temperature sensor [13].

As a result, numerous studies have been performed in a broad range of nanostructures, promoting significant advances in the performance of such effects [4,14,15].

Within this field, Anomalous Nernst (ANE) and the Longitudinal Spin Seebeck (LSSE) effects have distinguished positions, emerging as interesting phenomena for biosensors and spin caloritronics devices [16,17,18]. Both effects generally consist in the application of a temperature gradient ∇T and a magnetic field $\vec{H}$, thus generating an electric field $\vec{E}$ that can be expressed as [19]

(1) $$\vec{E}=-S\left(\hat{m}\times \nabla T\right),$$

where $S=\lambda \mu_{\circ }m_{s}$, $\mu_{\circ }$ is the vacuum magnetic permeability, and $m_{s}$ is the saturation magnetization of the ferromagnetic alloy, which is oriented along to the unit vector $\hat{m}$. Here, λ is a fundamental coefficient that is straightly related to the nature of the involved effect. Specifically, it is common to assume $\lambda =\lambda_{N}$, i.e., the well-known Anomalous Nernst coefficient, for a sample consisting of a single ferromagnetic metallic layer in which ANE is the unique effect in the system. However, for a bilayer structure composed by a ferromagnetic metallic layer capped by a non-magnetic metal in turn, an effective $\lambda_{eff}$ coefficient shall be taken into consideration. Remarkably, for this latter, both ANE and LSSE contribute to the generation of the electric field $\vec{E}$.

In a typical LSSE experiment, the electric field associated with the effect is measured through the Inverse Spin Hall Effect (ISHE). Nevertheless, we may find in the literature several studies uncovering distinct ways to overcome this experimental adversity, split the contributions of the LSSE and ANE effects [5,20], or improve the thermoelectric conversion efficiency. For instance, very recently, Holanda and colleagues [20] proposed a Si/NiFe/NiO/Pt heterostructure to detect the ANE and LSSE thermoelectric voltages simultaneously, thus allowing the comparison of the results with the values acquired for a single Si/NiFe film. Following a distinct approach, aiming to obtain optimized responses, one may focus on bilayer structures and play with the choice of the ferromagnetic material and the metallic capping layer, both having deep impacts on the thermoelectric conversion efficiency. In this case, it is well known that capping-layer materials with high spin-orbit coupling are the main elected for investigations of spintronics effects [21]. This fact is justified given that they enable the interconversion between charge current and spin current [5]. Nonetheless, the ferromagnetic material has a fundamental role in this interconversion, being responsible for the spin current injection. Therefore, properties as magnetic permeability, magnetic anisotropy, damping parameter, and capability to generate spin-polarized current are key features that must be tuned to improve the thermoelectric effects in bilayer structures [22,23,24].

Within this spirit, Heusler alloys appear as attractive candidates for thermoelectric applications [25,26,27], given that in theory, they can present energy gap around the Fermi level, making possible the achievement of 100% spin-polarized currents [28]. Among the numerous Heusler alloys, Co${}_{2}$FeAl (CFA) is a conspicuous material due to its high magnetic permeability and controllable magnetic anisotropies [29,30], features often found in CFA films. Theoretically, this alloy has been widely studied through first-principle calculations [31,32,33,34]. Experimentally, the magnetic properties in CFA films are strongly dependent of parameters used during the production process, such as temperature deposition and annealing after the deposition [35], as well as on the employed substrate. Therefore, the control of the features of the CFA alloy as magnetic anisotropy [35,36,37,38], associated with the integration between electrical and magnetic properties, may open new roads to explore this material and to modulate its thermoelectric response.

In this work, we explore the possibility of modulating a thermoelectric effect by magnetic anisotropy. Specifically, we investigate the Longitudinal Spin Seebeck Effect in films with a CFA/W bilayer structure grown onto GaAs (100) substrate, systems with induced uniaxial magnetic anisotropy combined with cubic magnetic anisotropy. From numerical calculations, we address the magnetic behavior and thermoelectric response of the films. By comparing experiment and theory, we show that the thermoelectric voltage curves may be controlled by the association of magnetic anisotropy induction and experimental parameters employed in the LSSE experiment. These results enable us to modulate the thermoelectric response as a function of the applied magnetic field, opening new possibilities for sensor application.

## 2. Materials and Methods

Here, we perform experiments in a set of films with a CFA/W bilayer structure, in which the CFA layer in each sample was grown at a given temperature. Specifically, CFA layers were prepared at the selected temperatures of 300, 573, 673 and 773 K [35]. The thickness of the ferromagnetic and metallic non-magnetic layers in the bilayer structure is 53 and 2 nm, respectively. The films were deposited by magnetron sputtering onto GaAs (100) substrates with dimensions of 3×6 mm${}^{2}$, previously annealed at 923 K, and covered by a 2-nm-thick W buffer layer. The deposition process was carried out with the following parameters: base pressure of $7.8\times 10^{-8}$ Torr, Ar pressure during disposition of $3\times 10^{-3}$ Torr with flow of 14 sccm, current of 200 mA set in the DC source for the CFA layer deposition, and 140 mA set in similar source for the grown of the W layer. Using these parameters, we reach deposition rates were 0.6 nm/s and 0.53 nm/s for the CFA and W, respectively. During the whole production process for all samples, a constant 1.0 kOe magnetic field was applied perpendicularly to the main axis of the substrate.

The structural characterization of the samples was obtained by x-ray diffraction. The experiments were performed using CuK${}_{\alpha }$ radiation. While low-angle x-ray diffraction was employed to determine the deposition rate and calibrate the film thickness, high-angle x-ray diffraction measurements were used to verify the structural character of the samples.

Quasi-static magnetic characterization was obtained using a LakeShore Model 7404 Vibrating Sample Magnetometer (VSM). Specifically, the in-plane magnetic properties were verified through magnetization curves, acquired with a maximum magnetic field value of ±500 Oe. The set up allowed us the rotation of the sample. Thus, to verify the magnetic anisotropy induced in the films, we measured magnetization curves for the different φ values, the angle between the magnetic field and the shortest axis of the samples.

Ferromagnetic resonance (FMR) measurements were carried out with a Bruker EPR system operating at 9.838 GHz (TE011 mode). In this case, the sample was placed in the center of a cylindrical cavity, in which the microwave magnetic field is maximized, and the electrical field is minimized. The set up also allowed us the rotation of the sample and experiments were performed at different φ values. The magnetic field, applied in the film plane, varied from 2.0 up to 3.5 kOe. It was modulated in the amplitude of 0.1 Oe at 100 kHz. Due to the modulation, the FMR power absorption curve took the shape of an absorption derivative, which was fitted by a Lorentzian-derivative function to extract resonance field and linewidth.

**Figure 1:**
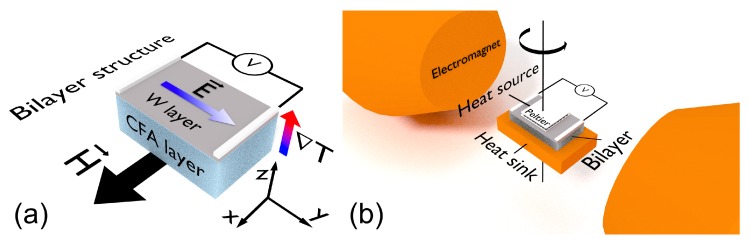
Schematic representation of our bilayer structure and the LSSE experiment. (**a**) Bilayer structure and definition of the coordinate system used in the numerical calculations. Given that φ is the angle between the magnetic field and the shortest axis of the sample, $\varphi =0^{\circ }$ is found when $\vec{H}$ is along the *x* axis. (**b**) LSSE experimental setup employed for the thermoelectric voltage measurements. The system allowed us the rotation of the sample during the experiment. Notice that the configuration represented in (**b**) corresponds to $\varphi =90^{\circ }$.

At last, we used a home-made Longitudinal Spin Seebeck Effect system (LSSE) to perform thermoelectric voltage measurements. Further information on the experimental system can be found in Ref. [39]. The system consists of a heat source (Peltier modulus) and a heat sink composed by a Cu block, in which the film is placed between them. The thermal conductivity was improved by the thermal paste, while the electrical contacts were made using silver paint. The system (heat sources and film) was connected to a step-motor, making possible the sample rotation and the measurement of the thermoelectric voltage at different φ values. Here, following the previous definition considered for the magnetic characterization, we kept the definition of φ as the angle between the magnetic field and the shortest axis of the sample. Figure 1 depicts a schematic representation of our bilayer structure and the LSSE experiment.

## 3. Results and Discussion

It is known that the structural features of films have a strong influence on their magnetic properties.

In ferromagnetic films produced onto amorphous substrates, a considerable out-of-plane anisotropy contribution is observed as the thickness is increased [40]. This behavior is related to the local stress storage in the film. On the other hand, by employing oriented substrates, we may promote a significant reduction of the local stored stress, favoring the appearance of magnetic anisotropy of magnetocrystalline nature.

This is precisely our intent in this work, in which we made use of an oriented substrate and annealing during the deposition to produce films having induced uniaxial magnetic anisotropy combined with cubic magnetic anisotropy.

### 3.1. Structural Results

**Figure 2:**
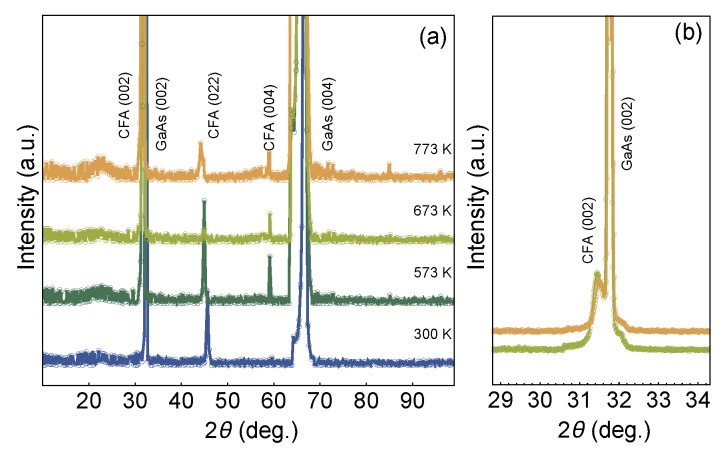
(**a**) High-angle x-ray diffraction results for the Co${}_{2}$FeAl/W bilayers grown onto GaAs (100) substrate. (**b**) Detailed view of the curves, in which the CFA(002) peak can be visualized. The CFA pattern is obtained from ICDD 01-0715670. Notice the polycrystalline character of the CFA alloy in all samples of the set.

Regarding the structural properties, Figure 2 shows the high-angle XRD results obtained for our set of films with CFA/W bilayer structure grown onto GaAs (100) substrate. First, the diffractograms disclose the (002) planes of the GaAs substrate [41,42,43], assigned by the well-defined and high-intensity peak located at $2\theta \approx 31.9^{\circ }$. Second, no evidence of the W layer is verified here, as expected, a fact that is associated with the reduced thickness of this layer. Going beyond, with respect to the CFA layer, the results clearly reveal a peak located at $2\theta \approx 44.73^{\circ }$ that is a fingerprint of the (022) texture of the CFA alloy, and peaks at $2\theta \approx 31.55^{\circ }$ and $59.15^{\circ }$, which are signatures of the CFA (002) texture. All these CFA peaks are identified from the pattern found in ICDD 01-0715670. Similar structural results have been previously reported in the literature for CFA films grown onto distinct oriented substrates [35,37]. In particular, the peaks observed here enable us to infer the polycrystalline character of the CFA alloy, with an A2 structure, suggesting disorder of the Co, Fe, and Al sites [38,44,45].

### 3.2. Magnetic Properties

**Figure 3:**
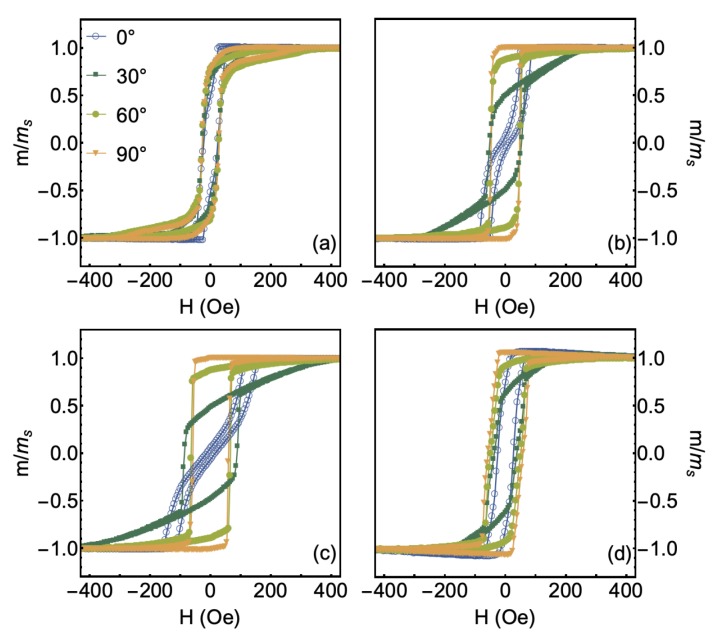
Normalized magnetization curves for the Co${}_{2}$FeAl/W bilayers grown onto GaAs (100) substrate, in which the CFA layer was deposited at the temperatures of (**a**) 300 K, (**b**) 573 K, (**c**) 673 K, and (**d**) 773 K. Here we present just the curves measured for $\varphi =0^{\circ }$, $30^{\circ }$, $60^{\circ }$ and $90^{\circ }$. All the samples of the set present uniaxial magnetic anisotropy combined with cubic magnetic anisotropy, although these features are more evident for the ones produced at the temperatures of 573 K and 673 K.

With respect to the magnetic properties, Figure 3 presents the magnetization curves measured for the CFA/W bilayer films at selected φ values. The angular dependence of the magnetization curves confirms the induction of magnetic anisotropy in all films. Notice the remarkable differences among the curves obtained at different φ values, especially for the samples produced with the temperatures of 573 K and 673 K. For all studied bilayers, the curve acquired at $\varphi =0^{\circ }$ suggests the existence of an intermediate magnetic axis along this direction. Furthermore, the results reveal an easy magnetization axis along the direction of $\varphi =90^{\circ }$, as well as a hard axis close to $\varphi =45^{\circ }$ (This latter is clearly observed through the FMR measurements, as we will see in the following). At last, no evidence of an out-of-plane anisotropy contribution is verified in the curves. Our results also corroborate previous studies reported in the literature, in which the CFA films were grown onto oriented substrates, such as Si, MgO, and GaAs [16,17,35]. All these features uncover the combination of induced uniaxial magnetic anisotropy and cubic magnetic anisotropy in the bilayers [46].

### 3.3. Modeling a System with Uniaxial Magnetic Anisotropy Combined with Cubic Magnetic Anisotropy

Given all the state above, we took into account a modified Stoner–Wohlfarth model [39,47] to mimic our Co${}_{2}$FeAl/W bilayers having induced uniaxial magnetic anisotropy combined with cubic magnetic anisotropy. For the numerical calculations, we considered a system with 50 non-interacting magnetic domains, in which the free energy density for each domain is described by

(2) $$\begin{array}{c} \xi_{i}=-\vec{m_{i}}\cdot \vec{H}+4\pi m_{si}^{2}{\left(\hat{m_{i}}\cdot \hat{n}\right)}^{2}-k_{ui}{\left(\hat{m_{i}}\cdot {\hat{u}}_{ki}\right)}^{2}-\frac{1}{4}\left(\xi_{c1i}+\xi_{c2i}\right). \end{array}$$

Here, the first term is associated with the Zeeman interaction, the second one describes the shape anisotropy, the third tells on the uniaxial magnetic anisotropy, and the last term is related with the magnetocrystalline anisotropy. Furthermore, the considered quantities are defined as the following: $\hat{m_{i}}$ is the unit vector of the magnetization, $m_{si}$ is the saturation magnetization, $\vec{H}$ is the magnetic field, $\hat{n}$ the unit vector normal to the film plane ($\theta_{n}=0^{\circ }$), and $k_{ui}=\frac{m_{si}h_{ki}}{2}$, where $h_{ki}$ is the anisotropy field related to the uniaxial magnetic anisotropy oriented along ${\hat{u}}_{ki}$ for each domain. At last, for the cubic magnetic anisotropy energy density,

(3) $$\xi_{c1i}=k_{c1i}\left(\alpha_{1i}^{2}\alpha_{2i}^{2}+\alpha_{1i}^{2}\alpha_{3i}^{2}+\alpha_{2i}^{2}\alpha_{3i}^{2}\right),$$

and

(4) $$\xi_{c2}=k_{c2}\left(\alpha_{1i}^{2}\alpha_{2i}^{2}\alpha_{3i}^{2}\right)$$

where $\alpha_{1i}$, $\alpha_{2i}$, and $\alpha_{3i}$ are the components of the unit vector of the magnetization, $\alpha_{1i}=\cos\varphi_{mi}\sin\theta_{mi}$, $\alpha_{2i}=\sin\varphi_{mi}\sin\theta_{mi}$ and $\alpha_{3i}=\cos\theta_{mi}$.

For the case in which the uniaxial anisotropy lies in the film plane and the magnetic field is also applied plane, Equation (2) is drastically simplified and can be rewritten as

(5) $$\begin{array}{c} \xi_{i}=-m_{si}\;H\;\cos\left(\varphi -\varphi_{mi}\right)-k_{ui}\;\sin{\left(\varphi_{ki}-\varphi_{mi}\right)}^{2}-\frac{1}{4}k_{c1i}\left(\cos{\left(\varphi_{mi}\right)}^{2}\sin{\left(\varphi_{mi}\right)}^{2}\right). \end{array}$$

From the minimization of Equation (5) for a given magnetic field $\vec{H}$, we are able to find the equilibrium angle of the magnetization of the i–th domain. This angle is a result of the competition between the contributions of the magnetic field and the magnetic anisotropies in the system. To account the whole magnetic behavior, in turn, we also need to consider the magnetic anisotropy dispersion of the system. Further information on the numerical calculations will be provided in the next sections, combining theory and experiment.

### 3.4. Magnetic Response at Saturated State

**Figure 4:**
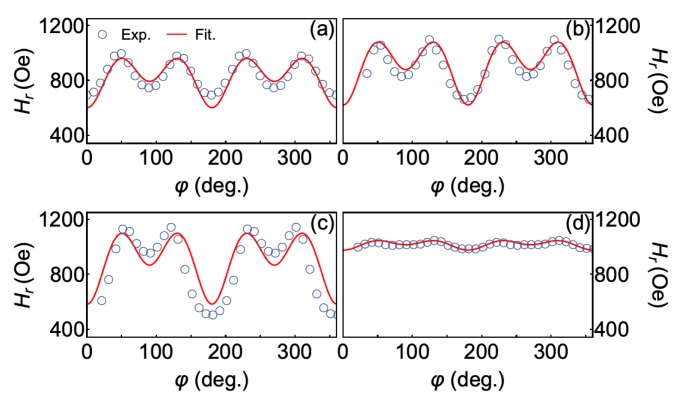
Angular dependence of the resonance field $H_{r}$ obtained from the FMR absorption derivative signal measured for the Co${}_{2}$FeAl/W bilayers in which the CFA layer was grown at (**a**) 300 K, (**b**) 573 K, (**c**) 673 K, and (**d**) 773 K. The red lines are fittings obtained with Equations (6) and (5).

Moving forward, evidence of the combination of anisotropies was also investigated through ferromagnetic resonance experiments. In particular, angular FMR measurements enable us to confirm the observed anisotropies, as well as to infer their relative contribution to the whole magnetic behavior. From the FMR absorption derivative signal obtained in the experiments for our samples, not shown here, we determined the linewidth ΔH and resonance field $H_{r}$, this latter presented in Figure 4.

From a general point of view, all bilayer films share the very same angular dependence of the resonance field $H_{r}$. In particular, the curves are characterized by four peaks with similar amplitude and four valleys, which are split in two groups according to the amplitude values. This behavior is a clear signature of the uniaxial magnetic anisotropy combined with cubic magnetic anisotropy. Furthermore, as expected, we confirm here that the samples produced with the temperatures of 573 K and 673 K do have more intense magnetic anisotropies, which are depicted by the larger amplitude variations of $H_{r}$.

In order to obtain further information of our system and estimate anisotropy constants, we considered the well-known angular resonance frequency $\omega_{r}$ and linewidth of the resonance absorption Δω, which can be written as [24,48,49]

(6) $$\omega_{r}=\frac{\gamma }{m_{s}\sin\theta_{m}}\sqrt{1+\alpha^{2}}\sqrt{\xi_{\theta \theta }\xi_{\varphi \varphi }-\xi_{\theta \varphi }^{2}}\;,$$

and

(7) $$\Delta \omega =\frac{\alpha \gamma }{m_{s}}\left(\xi_{\theta \theta }+\frac{\xi_{\varphi \varphi }}{\sin^{2}\theta_{m}}\right).$$

Here, $\gamma =|\gamma_{G}|/\left(1+\alpha^{2}\right)$, in which $\gamma_{G}$ is the gyromagnetic ratio and α the damping parameter; and $\xi_{\theta \theta }$, $\xi_{\varphi \varphi }$, $\xi_{\varphi \theta }$, and $\xi_{\theta \varphi }$ are the second derivatives of the magnetic free energy density, defined by the magnetization vector oriented by the angles $\theta_{m}$ and $\varphi_{m}$, at a given magnetic field.

Then, we performed fittings of the angular dependence of the resonance field $H_{r}$ using Equation (6) and taking into account the free energy density for a system with uniaxial magnetic anisotropy combined with cubic magnetic anisotropy, i.e., Equation (5). The magnetic parameters $m_{s}$, $k_{u}$ and $k_{c1}$ obtained from the fits of the FMR data are summarized in Table 1.

### 3.5. Experimental Results for Thermoelectric Effect

Based on the experimental results obtained in the structural and magnetic characterizations so far, from now, we focus our attention on the thermoelectric voltage measurements performed in the Co${}_{2}$FeAl/W bilayers in which the CFA layer was deposited at the temperatures of 573 K and 673 K.

**Figure 5:**
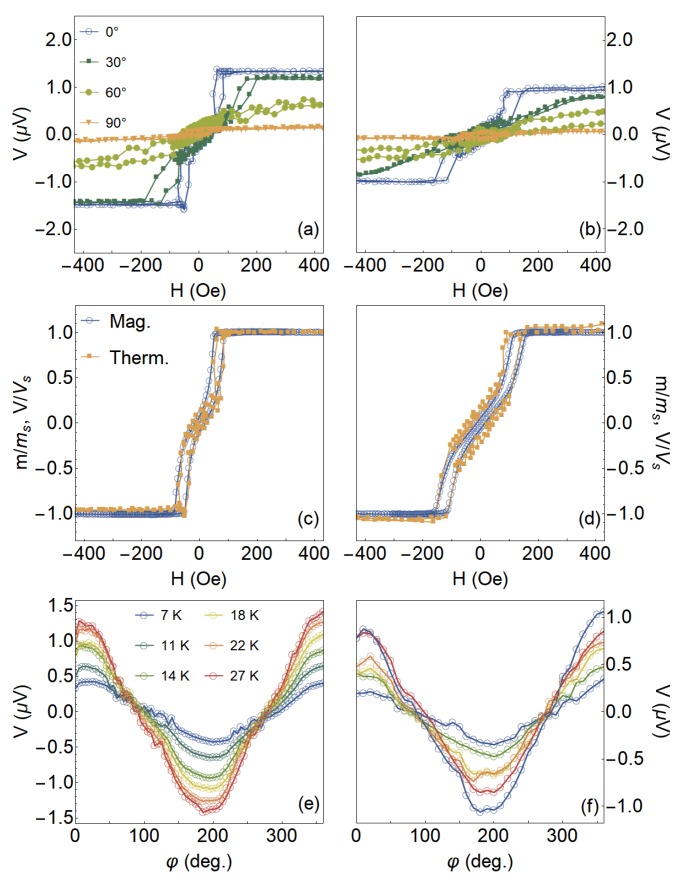
Experimental thermoelectric effect results. Thermoelectric voltage as a function of the magnetic field, at selected φ values and setting ΔT=27 K, for the (**a**) CFA/W bilayers in which the CFA layer was grown at (**a**) 573 K and (**b**) 673 K. (**c**,**d**) Comparison of the shape of normalized magnetization and thermoelectric voltage curves acquired at $\varphi =0^{\circ }$, with ΔT=27 K, for these samples. (**e**,**f**) Angular dependence of the thermoelectric voltage at H=+500 Oe for distinct ΔT values for the very same samples.

Figure 5 shows the experimental results of the magnetic response and the thermoelectric voltage for both samples. Specifically, Figure 5a,b presents the thermoelectric voltage as a function of the magnetic field, at selected φ values and setting ΔT=27 K. Notice the quite-interesting evolution in the shape of the curves as the magnitude and orientation of the field are altered. The one measured at $\varphi =0^{\circ }$ leads to a field configuration in which the thermoelectric voltage has a shape similar to that presented in the magnetization characterization, as we can clearly confirm from the plots shown in Figure 5c,d. However, with the increase of the φ, we verify a decrease of the thermoelectric voltage at saturation, leading to the suppression of the signal at $\varphi =90^{\circ }$, as expected. It is worth remarking that the overall thermoelectric voltage response is a result of the combination of effects associated with magnetic anisotropies in the samples and the thermoelectric voltage measurement configuration employed in the experiment [39].

Figure 5e,f shows *V* behavior as a function of φ, at H=+500 Oe, for selected ΔT values. At this field value, our system is magnetically saturated, in a sense that the magnetization follows the orientation of the magnetic field. As expected, the curves draw a clear dependence of *V* with φ, shown by a well-defined cosine shape. Furthermore, although the angular dependence is kept constant, the amplitude of the curves is altered with ΔT. All these features are in concordance with our theoretical predictions, discussed in the next sections.

### 3.6. Theoretical Approach for Thermoelectric Effect

As aforementioned, for ANE and LSSE effects, the application of a temperature gradient ∇T and a magnetic field $\vec{H}$ generates an electric field $\vec{E}$ given by Equation (1). Based on our experimental findings, for our theoretical approach, we considered a typical experiment in a film, in which the temperature gradient is normal to the film plane, while the magnetization lies in the plane, as depicted in Figure 1a.

The corresponding thermoelectric voltage, detected by electrical contacts at the ends of the main axis of the film, is thus given by

(8) $$V=-\int_{0}^{L}\vec{E}\cdot d\vec{l},$$

where *L* is the distance between the electrical contacts and $d\vec{l}=dl\hat{j}$ in our case.

Then, using Equations (1) and (8), we obtain

(9) $$V=\lambda \mu_{\circ }L\Delta T_{f}m_{s}cos\varphi_{m}.$$

In this case, $\Delta T_{f}$ is the temperature variation across the bilayer, which is related with temperature variation ΔT measured experimentally across the sample [19,39],

(10) $$\Delta T_{f}=\frac{t_{f}K_{sub}}{t_{sub}K_{f}}\Delta T,$$

where $K_{sub}$ and $t_{sub}$ are the thermal conductivity and thickness of the substrate, while $K_{f}$ and $t_{f}$ are the respective quantities for the CFA alloy. Here, we assumed $K_{sub}=55$ W/Km and $t_{sub}=0.7$ mm, while $K_{f}=129$ W/Km and $t_{f}=55$ nm for the CFA layer.

Notice that the *V* response given by Equation (9) is straightly dependent on the magnetic state of the sample through $\varphi_{m}$, which in turn is a result of the minimization process of free energy density of the system. This latter brings information on the magnetic field and the magnetic anisotropies in the system, given by Equation (5) in our case.

### 3.7. Numerical Calculations and Comparison between Theory and Experiment

We performed numerical calculations for two magnetic systems, distinct just with respect to the magnetic anisotropy dispersion. Remember that our system has a combination of induced uniaxial magnetic anisotropy and cubic magnetic anisotropy. Then, first, we calculated the magnetic and thermoelectric properties for a system without magnetic anisotropy dispersion, with $m_{si}=m_{s1}=\ldots =m_{s50}=m_{s}=1330$ G, $k_{ui}=k_{u1}=\ldots =k_{u50}=k_{u}$, with $h_{k}=220$ Oe and $\varphi_{ki}=\varphi_{k1}=\ldots =\varphi_{k50}=0^{\circ }$ (*x* direction in Figure 1a), as well as $k_{c1i}=k_{c11}=\ldots =k_{c150}=2.8\;k_{u}$. In a second moment, to mimic a magnetic system with a given uniaxial anisotropy dispersion, we considered the very same parameters, but with the fundamental difference of having $\varphi_{ki}$ dispersed linearly around $0^{\circ }$, with dispersion range of $\Delta \varphi_{k}=10^{\circ }$.

**Figure 6:**
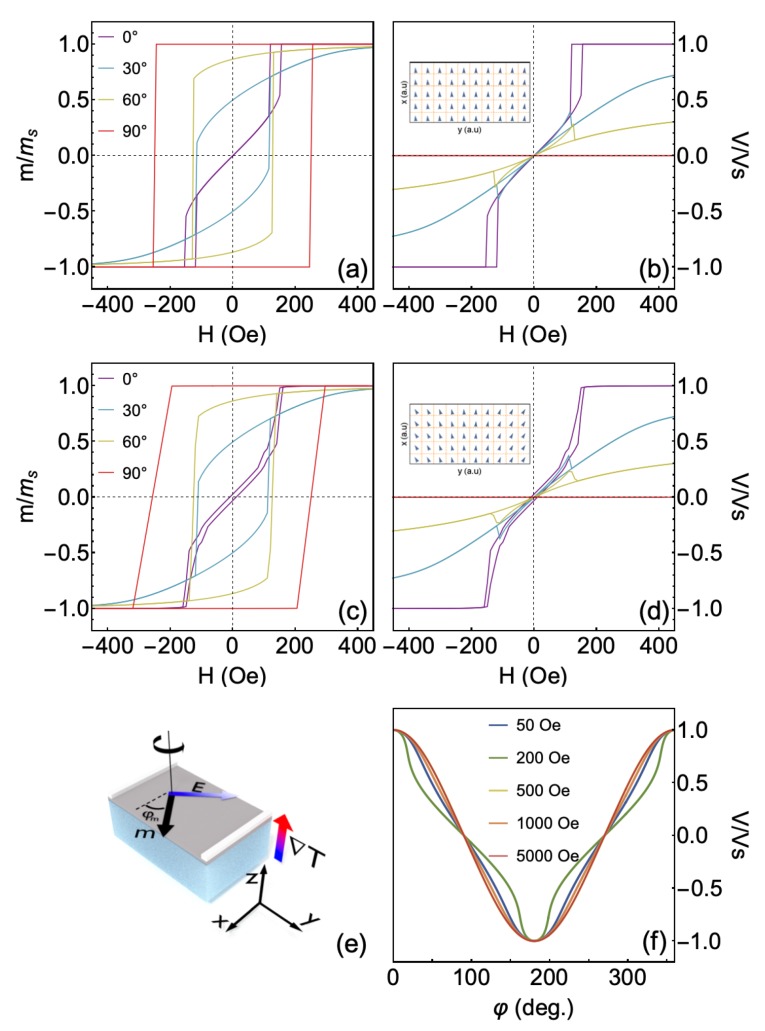
Numerical calculations of magnetic properties and thermoelectric voltage. (**a**) Normalized magnetization and (**b**) thermoelectric voltage curves, at selected φ values, for a system having induced uniaxial magnetic anisotropy and cubic magnetic anisotropy, without anisotropy dispersion. The calculations were performed with $m_{si}=m_{s1}=\ldots =m_{s50}=m_{s}=1330$ G, $k_{ui}=k_{u1}=\ldots =k_{u50}=k_{u}$, with $h_{k}=220$ Oe and $\varphi_{ki}=\varphi_{k1}=\ldots =\varphi_{k50}=0^{\circ }$, as well as $k_{c1i}=k_{c11}=\ldots =k_{c150}=2.8\;k_{u}$. Furthermore, we considered ΔT=27 K. In (**b**), the inset illustrates a simple view of magnetic domains in the system without anisotropy dispersion. (**c**,**d**) Similar plots for the the same system having uniaxial anisotropy dispersion, in which $\varphi_{ki}$ dispersed linearly around $0^{\circ }$, with dispersion range of $\Delta \varphi_{k}=10^{\circ }$. In (**d**), the inset also brings the simple view of the magnetic domains in the system without anisotropy dispersion. (**e**) Schematic representation of our bilayer structure in the experiment, in which we depict $\varphi_{m}$ that is inserted in Equation (9) for the calculations. (**f**) Angular dependence of the thermoelectric voltage at ΔT=27 K for distinct field values for the system with anisotropy dispersion. The curves are normalized in order to make easier the direct comparison between results.

Figure 6 shows the numerical calculations of the magnetic response and the thermoelectric voltage for systems having induced uniaxial magnetic anisotropy and cubic magnetic anisotropy, without and with uniaxial anisotropy dispersion.

For the system without anisotropy dispersion (Figure 6a,b), we verify that the magnetization curve calculated for $\varphi =0^{\circ }$ reflects the behavior of an intermediate magnetic axis, a result of the competition between the uniaxial and cubic magnetic anisotropies. Furthermore, as expected, the easy and hard magnetization axes are aligned to $\varphi =90^{\circ }$ and $45^{\circ }$, respectively [46]. This behavior is in concordance with the experimental results previously discussed in Section 3.2 and Section 3.4. Regarding the thermoelectric calculations, the results obtained for the $\varphi =0^{\circ }$ leads to a curve with a similar shape to that one obtained in the magnetization calculation. With the increase of φ, we confirm the decrease in the thermoelectric voltage at saturation, reaching zero for $\varphi =90^{\circ }$. These features are in perfect agreement with our discussion presented in Section 3.5.

When the magnetic anisotropy dispersion is inserted in the system (Figure 6c,d), the overall effective magnetic anisotropy is not strongly altered. As a consequence, the shapes of the curves in the magnetization response and thermoelectric voltage are globally kept. However, remarkably, the anisotropy dispersion refines the model, introducing details of the magnetic behavior that are fundamental to the description of real systems. Specifically, the inclusion of the anisotropy dispersion leads to smoother magnetization variations and is responsible for modifications in the magnetic permeability, coercive field and remanent magnetization. All these changes yield deep impacts in the thermoelectric voltage response.

As aforementioned, the thermoelectric voltage response is a combination of effects associated with magnetic anisotropies in the samples and the thermoelectric voltage measurement configuration employed in the experiment. In some sense, it is reasonable its dependence with φ (Figure 6e). Therefore, we also perform numerical calculations of the *V* behavior as a function of φ for the system with anisotropy dispersion, at distinct *H* values, shown in Figure 6f. As expected, the curves of the thermoelectric voltage are strongly dependent on both the φ angle and the amplitude of the magnetic field *H*. The equilibrium angle of magnetization, $\varphi_{m}$ obtained from the minimization of Equation (5), is the result of the competition between the energy density contributions of magnetic induced uniaxial and cubic anisotropies with the Zeeman energy density. For field values high enough to saturate the system magnetically, the angular dependence of *V* is in perfect concordance with Equation (9), given by a cosine of φ, given $\varphi_{m}=\varphi$. However, for unsaturated states, the cosine behavior is lost, bringing information on the magnetic anisotropy and anisotropy field.

It is interesting to notice that the previous calculations have qualitatively described the main features of the magnetic behavior and thermoelectric voltage response in a system having induced uniaxial magnetic anisotropy combined with cubic magnetic anisotropy.

**Figure 7:**
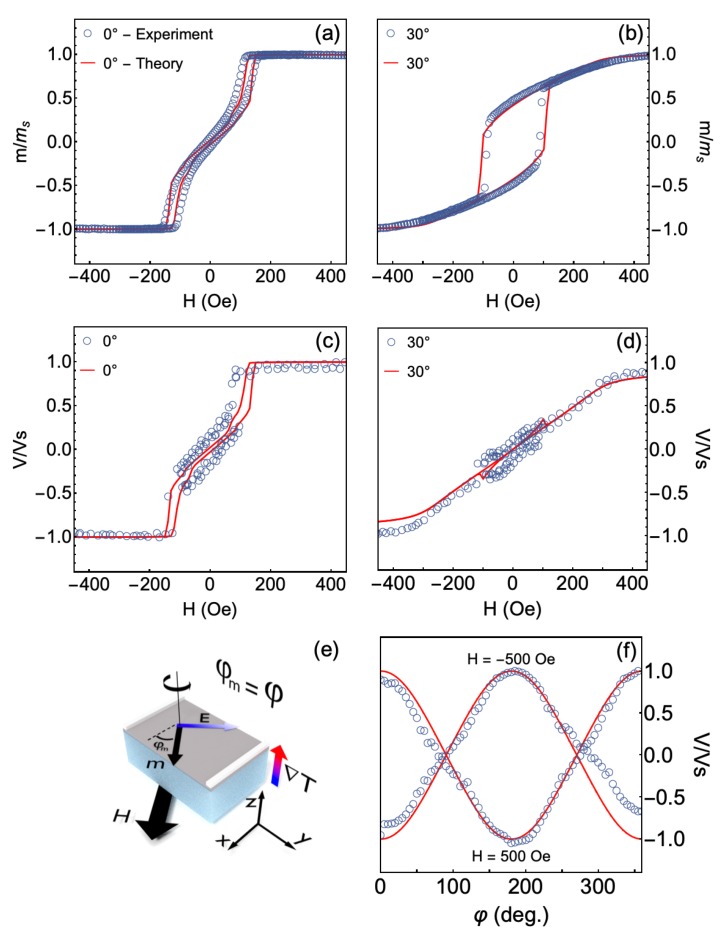
Comparison between theory and experiment: Normalized magnetization curves, at (**a**) $\varphi =0^{\circ }$ and (**b**) $30^{\circ }$, for the CFA/W bilayer in which the CFA layer was grown at 573 K. (**c**,**d**) Corresponding normalized thermoelectric voltage curves. (**e**) Schematic representation of our bilayer structure in the experiment, in which we depict $\varphi_{m}$ that is inserted in Equation (9) for the calculations. (**f**) Normalized curves of the angular dependence of the thermoelectric voltage, at ΔT=27 K, for H=±500 Oe. In particular, the calculations here were performed system with uniaxial anisotropy dispersion, with $m_{si}=m_{s1}=\ldots =m_{s50}=m_{s}=1330$ G, $k_{ui}=k_{u1}=\ldots =k_{u50}=k_{u}$, where $h_{k}=220$ Oe and $\varphi_{ki}$ is dispersed linearly around $0^{\circ }$, with dispersion range of $\Delta \varphi_{k}=10^{\circ }$, as well as $k_{c1i}=k_{c11}=\ldots =k_{c150}=2.8\;k_{u}$.

However, the most striking findings here are shown in Figure 7. Here we directly compare theory and experiment and uncover the evolution of the magnetic response and thermoelectric voltage with the amplitude and direction of the magnetic field. Furthermore, we corroborate the major role of the magnetic anisotropy dispersion to the description of the magnetic behavior and thermoelectric effect.

Notice the remarkable agreement between experiment and theory for the different conditions of measurement. Specifically, we disclose experimental results for the CFA/W bilayer in which the CFA layer was grown at 573 K. For the numerical calculations, we considered a system with uniaxial anisotropy dispersion, with $m_{si}=m_{s1}=\ldots =m_{s50}=m_{s}=1330$ G, $k_{ui}=k_{u1}=\ldots =k_{u50}=k_{u}$, where $h_{k}=220$ Oe and $\varphi_{ki}$ is dispersed linearly around $0^{\circ }$, with dispersion range of $\Delta \varphi_{k}=10^{\circ }$, as well as $k_{c1i}=k_{c11}=\ldots =k_{c150}=2.8\;k_{u}$. Remember that these magnetic parameters are the very same used for the previous calculations shown in Figure 6, all of them obtained from the magnetic characterization discussed in Section 3.2 and Section 3.4. Therefore, from the comparison, we confirm the validity of our theoretical approach, including the description of our system given by Equation (5), as well as we corroborate magnetic parameters as the anisotropy constants and the saturation magnetization, presented in Table 1.

Hence, we were able to describe through the numerical calculations all the main features of the magnetic behavior and thermoelectrical effect in a magnetic system having induced uniaxial magnetic anisotropy combined with cubic magnetic anisotropy. From a general point of view, we highlight that this theoretical approach may be modified to describe any magnetic system if considered the appropriate free energy density. Furthermore, here, we showed the possibility of modulating the LSSE thermoelectric effect by the magnetic anisotropy induction. Specifically, the thermoelectric effect, measured through LSSE, emerged as a powerful tool to investigate fundamental parameters of a magnetic system, such as anisotropy configuration and anisotropy dispersion.

## 4. Conclusions

In conclusion, we investigated the Longitudinal Spin Seebeck Effect in films with a CFA/W bilayer structure grown onto GaAs (100) substrate. We verified that the magnetic properties of the CFA films are strongly dependent of parameters used during the production process, such as temperature deposition and annealing after the deposition. By setting optimal conditions, we manufactured systems having induced uniaxial magnetic anisotropy combined with cubic magnetic anisotropy. We observed clear dependence of the thermoelectric voltage curves with the magnetic anisotropy and experimental parameters employed in the LSSE experiment. By comparing experiment and theory, we confirmed the possibility of modulating a thermoelectric effect by magnetic anisotropy in Co${}_{2}$FeAl Heusler alloy. Our results raise important features, contributing to the integration between electrical and magnetic properties that may promote improvements of the thermoelectric response in such nanostructures. These results enabled us to modulate the thermoelectric response as a function of the external magnetic field, an essential feature for sensor applications.

## Figures and Tables

**Table 1:** Parameters obtained from the fits of the experimental FMR data for our set of Co${}_{2}$FeAl/W bilayers. For the fits, we assumed $\alpha \approx 2\times 10^{-3}$ and γ=2.8 MH/Oe.

CFA Grown Temperature (K)	$m_{s}$ (emu/cm${}^{3}$)	$k_{u}$ (ergs/cm${}^{3}$)	$k_{c1}$ (ergs/cm${}^{3}$)
300	1340	$1.00\times 10^{5}$	$3.01\times 10^{5}$
573	1330	$1.33\times 10^{5}$	$3.72\times 10^{5}$
673	1330	$1.66\times 10^{5}$	$4.82\times 10^{5}$
773	1328	$1.97\times 10^{5}$	$5.53\times 10^{4}$
